# A novel k-mer mixture logistic regression for methylation susceptibility modeling of CpG dinucleotides in human gene promoters

**DOI:** 10.1186/1471-2105-13-S3-S15

**Published:** 2012-03-21

**Authors:** Youngik Yang, Kenneth Nephew, Sun Kim

**Affiliations:** 1 J Craig Venter Institute, San Diego, CA, USA; 2 Medical Sciences Program, Indiana University School of Medicine, Bloomington, IN, USA; 3 School of Computer Science and Engineering, Bioinformatics Institute, Interdisciplinary Program in Bioinformatics, Seoul National University, Seoul, Korea

## Abstract

**Background:**

DNA methylation is essential for normal development and differentiation and plays a crucial role in the development of nearly all types of cancer. Aberrant DNA methylation patterns, including genome-wide hypomethylation and region-specific hypermethylation, are frequently observed and contribute to the malignant phenotype. A number of studies have recently identified distinct features of genomic sequences that can be used for modeling specific DNA sequences that may be susceptible to aberrant CpG methylation in both cancer and normal cells. Although it is now possible, using next generation sequencing technologies, to assess human methylomes at base resolution, no reports currently exist on modeling cell type-specific DNA methylation susceptibility. Thus, we conducted a comprehensive modeling study of cell type-specific DNA methylation susceptibility at three different resolutions: CpG dinucleotides, CpG segments, and individual gene promoter regions.

**Results:**

Using a k-mer mixture logistic regression model, we effectively modeled DNA methylation susceptibility across five different cell types. Further, at the segment level, we achieved up to 0.75 in AUC prediction accuracy in a 10-fold cross validation study using a mixture of k-mers.

**Conclusions:**

The significance of these results is three fold: 1) this is the first report to indicate that CpG methylation susceptible "segments" exist; 2) our model demonstrates the significance of certain k-mers for the mixture model, potentially highlighting DNA sequence features (k-mers) of differentially methylated, promoter CpG island sequences across different tissue types; 3) as only 3 or 4 bp patterns had previously been used for modeling DNA methylation susceptibility, ours is the first demonstration that 6-mer modeling can be performed without loss of accuracy.

## Background

DNA methylation is the chemical modification of DNA bases, mostly on cytosines that precede a guanosine in the DNA sequence, i.e., the CpG dinucleotides. This epigenetic modification involves the addition of a methyl group to the number 5 carbon of the cytosine pyrimidine ring. DNA methylation is essential for cellular growth, development and differentiation [1], playing a fundamental role in the activation of genes at the transcriptional level. In cancer cells, aberrant DNA methylation patterns, such as genome-wide hypomethylation and region-specific hypermethylation, are frequently observed [2]. CpG islands, short CpG-rich regions of DNA often located around gene promoters and normally protected from DNA methylation, become hypermethylated in cancer, contributing to transcriptional silencing [3,4]. As CpG island methylation patterns have been shown to differ across cancer types, recent studies have revealed that some CpG islands are "methylation sensitive", while others are "resistant" to DNA methylation [5]. Recent technological breakthroughs allow, for the first time, the capability to measure human methylomes at base resolution [6], providing unprecedented opportunities for understanding the phenomenon of methylation susceptibility.

### Previous work

Several recent studies have attempted to predict CpG island methylation patterns in normal and cancer cells. DNA pattern recognition and supervised learning techniques were used by Feltus et al to discriminate methylation-prone (MP) and methylation-resistant (MR) CpG islands based on seven DNA sequence patterns [7]. McCabe et al then developed a classifier (PatMAn) based on the frequencies of those seven patterns in cancer [8], followed by "SUPER-PatMAn" for predicting methylation susceptible CpG islands using both local sequence context and transacting factors such SUZ12 [9]. In addition, Feltus et al used motifs related to 28 MP and MR CpG islands to predict DNA methylation susceptibility [10], and Keshet et al showed evidence of instructive mechanisms in cancer cells, finding common sequence motifs in the regions of promoters whose genes show tumor-specific "methylation susceptibility" [11]. A prediction method for finding a minority class in an imbalanced data setting (which is the case for DNA methylation data), called "cluster_boost", was recently developed by Goh et al and used to identify novel hypermethylated genes in cancer [12]. Fang et al developed "MethCGI" to predict the methylation status of CpG islands using a support vector machine and both local sequence context and transcription factor binding sites [13]. Finally, a prediction method using DNA sequence features of various types, including sequence, repeats, predicted structure, CpG islands, and genes, was developed by Bock et al to predict binding sites, conservation, and single nucleotide polymorphisms [14].

While the focus of the above studies was on CpG island methylation susceptibility, recent experiments have convincingly demonstrated that methylation levels of CpG sites, i.e. genomic location of CpG dinucleotides, within a CpG island can be highly variable. For example, Handa et al found that certain sequence features flanking CpG sites were associated with high- and low-methylation CpG sites in an in vitro DNMT1 overexpression model [15]. Moreover, at single base pair resolution, Zhang et al demonstrated that DNA methylation levels frequently differ within a CpG island [16]. To investigate the role of DNA methylation during development in human embryonic stem cells Brunner et al developed Methyl-seq, which assays DNA methylation at more than 90,000 regions throughout the genome [17]. Using bisulfite sequencing data, Lister et al determined the first genome-wide, single-base-resolution maps of methylated cytosines in mammalian genomes (human embryonic stem cells (ESC) and fetal broblasts) [18]. By using "ultradeep" sequencing data from Taylor et al [19], we demonstrated that CpG flanking sequences can be used to model methylation susceptible CpG sites [20]. Finally, Previti et al analyzed tissue-specific CpG island methylation status, in terms of profiles created by probabilistically combining two sources of independent clusters (clusters from methylation data in 12 tissues and clusters from CGIs attributes) to demonstrate the predictive power of their method with a decision tree classifier [21]. Those investigators categorized profiles into four classes: *constitutive unmethylated, constitutive methylated, unmethylated in sperm, and differentially methylated *[21].

### Motivation

Previous CpG island methylation susceptibility prediction studies have not considered cell type-specific methylation status. Considering variations in DNA methylation level even in the same genomic regions of different types of cells, we asked the question: can cell type-specific DNA methylation susceptibility be modeled? The significance of exploring this question is based on evidence supporting the strong association of genomic sequence features with DNA methylation status. Furthermore, recent studies strongly indicate the existence of methylation sensitive/resistant CpG islands in different cancer types [5]. In this paper, we performed a comprehensive DNA methylation susceptibility modeling study in five different cell lines at three different levels: CpG sites, entire promoter regions, and short DNA segments. We focused on DNA methylation in the context of CpG dinucleotides in adult cells (we are aware of a recent study [18] reporting non-CpG methylation in ESC).

## Methods

### The problem: methylation susceptible dna segment modeling problem

#### The need for segment modeling

Bisulfite sequencing data clearly demonstrates that methylation levels, even within a single gene promoter, can be highly variable. Furthermore, a figure in Additional file 1 shows highly variable methylation of the same promoter sequence in five different cell lines, i.e. cell type-specific DNA methylation susceptibility (bisulfite sequencing data obtained from [16]).

#### Definition of the problem

The following notations were used to formally define the problem. A small set of pre-selected k-mers **x **= {*x_i_*}, where a k-mer is fixed number of DNA base pairs. Labels **t **= {*t_j_*} on data are assigned as +/- depending on methylation level *p_j _*of each sample.

For each cell type, a k-mer mixture logistic regression model (equation 1) was built using a small set of pre-selected patterns, i.e. *k*-mers. To select the best logistic model, predicted methylation at a CpG site (based on the logistic model under consideration) was compared with actual CpG methylation obtained from the bisulfite sequencing data. To make the comparison, we calibrated the predicted methylation level between 0 and 1 (below).

(1) $$y=\frac{1}{1+e^{-f\left(x\right)}}$$

where $f\left(x\right)=\sum_{i}\beta_{i}x_{i}$ and *β_i_*'s are parameters to be learned for the machine learning predictor.

##### The k-mer mixture modeling problem

Our goal was to test whether methylation susceptibility can be modeled by a logistic regression model using a small set of k-mers. Although using k-mers for DNA methylation modeling is not entirely new, to our knowledge, only short k-mers (3 or 4 bp in length) were used in previous studies [14]. As short k-mers can occur in almost every DNA sequence, modeling using 3 or 4 bp relies on k-mer frequency.

1. First, we attempted to use longer k-mers (up to 6 bp) to utilize those that only occur in methylation susceptible sequences (vs. frequency for short k-mers, described above).

2. Our goal of determining whether machine learning predictors can be built by using k-mers required that we address two important issues: over-fitting and generalizability of prediction beyond the test data. The over-fitting problem was addressed by selecting a small number of k-mers from the training data set (using a larger number of k-mers can easily over-fit the training data). The cross validation technique was used to test the generalizability of prediction power. We selected k-mers and built machine predictors by using only the training data set. We then assessed the predictor on the test data set not used for either selecting k-mer features or building predictors.

#### Two k-mer feature selection methods

We used a selected set of k-mers for DNA methylation susceptibility modeling in the different cell types. The research question explored in this paper is the feasibility of modeling methylation susceptible segments given a set of k-mers. As selection of the "best set" of k-mers for modeling was not explored (a solution to the combined problem was too difficult), we used two standard pattern selection methods for a two-class data set.

1. Feature selection with t-test: A popular t-test method was used to select k-mers because of its simplicity and applicability for all modeling approaches. For each attribute *a*, occurrences of *a *were counted in positive samples and negative samples. Then, the P-value of *a *was measured by t-test. A fixed number of patterns was selected from a list of k-mers ordered by P-value. Alternatively, patterns with a P-value below a threshold were selected.

2. Feature selection with the random forest technique: The RF algorithm [22] can be used for feature selection. The usefulness of the RF-based feature selection method was clearly demonstrated by Yi-Wei Chen and Chih-Jen Lin at the NIPS 2003 feature selection challenge [23]. We used an extended version of the RF-based feature selection method. Multiple rounds of the RF-based feature selection were performed using a balanced data set of methylation-susceptible and non-susceptible sequences. We performed *k *times of RF runs, where each RF run used *n *random trees; only top *N *attributes with z-scores > 0 were collected. After *k *RF runs, a subset of attributes, which had appeared *p*% times, were selected. The values were set *k *= 30, *n *= 100, *N *= 100, and *p *= 90 for the k-mer feature selection.

In both methods, we extracted a set of patterns in the balanced data set. First, centered at each CpG site, we extracted a flanking sequence of length *l*, where we set *l *= 100. A label of the CpG site was given as +/- depending on methylation level. Then, we balanced the data with even number of +/- classes. A set of all k-mers obtained in sliding windows on each sequence were used for k-mer feature selection.

#### Modeling methylation levels of DNA segments

##### Definition A boundary variable

**Figure 1:**
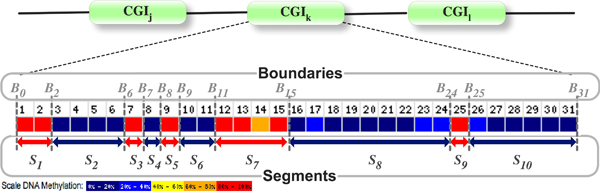
**Illustration of the initial segment definition.** Because all boundary variables are set to 1, 10 initial segments are defined. Later, the segment modeling algorithm considers alternative segment definition by changing the boundary variable values. Figure was modified from [16,25].

*B_i _*at a genomic sequence position is an indicator variable that is defined where two adjacent CpG sites have different labels. The value 1 of *B_i _*denotes that the genomic position is a boundary and the value 0 denotes that the position is not a boundary. A DNA segment *S *is defined by two boundary variables *B_a _*and *B_z _*where *B_a _*= 1 and *B_z _*= 1 and for all *a *<*i *<*z*, *B_i _*= 0. Figure 1 illustrates how boundary variables are used to define 10 segments. We call a set of DNA segments defined by the boundary variables a ***configuration***.

##### Labeling data

Given a segment *S_i_*, the methylation probability *p_i _*of a segment was defined as a ratio of the number of CpG sites with the + label to the number of CpG sites in the segment. Then, the label *t_i _*of *S_i _*was assigned + if *p_i _*is greater than 0.5. Otherwise, a label - was assigned to *t_i_*.

##### Attributes for modeling

K-mer occurrences in segments in the training data set were used as attributes. A small subset of k-mers features **x **was selected from all k-mers using the feature selection methods.

##### Modeling

A single logistic regression model was used to model all DNA segments for each cell line, using attributes **x **and labels **t**.

#### Segment-level modeling challenges: exponential search space

Although the methylation status of a DNA segment is defined by an aggregation of the methylation status of individual promoter regions (as we did for the whole promoter region-modeling approach), how to define methylation susceptible DNA segments is currently unknown. For example, consider a DNA segment with five CpG sites {*s*1, *s*2, *s*3, *s*4, *s*5} in a short DNA segment and assume that three sites, *s*1, *s*2, *s*4 are methylation susceptible and the other two sites *s*3, *s*5 are resistance methylation. By definition, the DNA segment is methylation susceptible, as the majority of sites (three) are methylation susceptible. However, if we divide the segment into two sub-segments, {*s*1, *s*2} and {*s*3, *s*4, *s*5}, there will be a segment that is susceptible to methylation and one that is resistant. To determine which of the two segment definitions can be better modeled for methylation susceptibility, enumeration of all possible definitions of segment configurations and for each definition of segment is required. We thus computed a "best fit" logistic model for methylation data in a cell line. The complexity of this problem can be discussed in terms of the well-known "counting the number of parenthesization" problem [24], because a parenthesis can define a segment of CpG sites. The number of parenthesis *P *(*n*) for *n *CpG sites is *P *(1) = 1; $P\left(n\right)=\sum_{i=1}^{n-1}P\left(i\right)P\left(n-i\right)$for *n *≥ 2. Given the complexity, an optimal solution using an exhaustive search algorithm is unlikely to be found (known to be Ω (2*^n^*) [24]). Thus, we developed a heuristic algorithm that used a random segment merging starting from the finest definition of segments.

### A random binary segment merging algorithm

A Naïve approach to segment modeling simply enumerates all possible segment configurations. Every combination of segment boundaries is considered, while changing the setting of values for boundary indicator variable *B_i _*∈ {0, 1}. Then, an error function for each segment set definition is computed. However, this requires the enumeration of a 2*^m ^*possible segment configurations, where *m *is the number of *B_i_*. To compute the optimal k-mer logistic regression model, segment boundaries must first be identified; however, as these are unknown, we started with an initial presumption of the methylation susceptible and resistant segments. We then used an iterative improvement procedure in search of both the segment definition and the best fitting logistic regression model. The major steps of the segment modeling algorithm are as follows:

1. **Initialization of a configuration: **Define a boundary variable *B_i _*= 1 at every genomic position where labels (+ or -) of two adjacent CpG sites around the position are different. Define a segment as a DNA region between two boundary variables set to 1. By taking this approach, we start with a configuration of smallest possible segments. By merging segments in many different ways and re-calculating the logistic regression model, the algorithm attempts to find the best segment configuration. This is how INITIALCONFIGURATION() is implemented in the HillClimbingConfigurationSearch in Algorithm 1.

2. **Computing a logistic regression model**: Given a k-mer occurrence and a segment configuration, compute a logistic regression model by (1). This is how COMPUTEMODEL() is implemented in the HillClimbingConfigurationSearch in Algorithm 1.

3. **Computing an error of a segment configuration**: Errors in the segment set S are measured by (2).

(2) $$O\left(S\right)=\overset{\left|S\right|}{\underset{i=1}{\sum }}w_{i}{\left({ŷ}_{i}-t_{i}\right)}^{2}$$

where |S| is the total number of segments, ${ŷ}_{i}$is the predicted methylation level of the segment *i*, *t_i _*is the actual methylation level of the segment *i*, and *w_i _*is the weight of each segment. A segment weight is defined as $w_{i}=\bar{\left|S\right|}/\left|S_{i}\right|$, where $\bar{\left|S\right|}$ is the average count of CpG sites in all segments and |*S_i_| *is the count of CpG in a segment. A weight of each segment *w_i _*is given as an inverse proportion to average segment size. In this way, large segments are penalized less, and vice versa. This is how COMPUTEERROR() is implemented in the HillClimbingConfigurationSearch in Algorithm 1.

#### The random binary segment merging algorithm

Given the current segment configuration {*B_i_*}, a segment is randomly chosen using a distribution of errors measured by a weighted square error. For a segment *B_j_*, the weighted square error is defined by $e_{j}=\beta_{j}{\left({ŷ}_{j}-t_{j}\right)}^{2}$ where the weight of the segment $\beta_{j}=\left|S_{i}\right|/\bar{\left|S\right|}$, ${ŷ}_{j}$ is the predicted methylation level of the segment *j*, and *t_j _*is the actual methylation level of the segment *j*. A segment is chosen by random sampling using a segment error vector <*e*_1_, . . . , *e*_n _> where *n *is the number of segments in the current segment configuration. The random sampling using a segment error vector <*e*_1_, . . . , *e_n _*> guides choosing a segment with a higher prediction error, but also ensure a random sampling. Note that segments that are already considered for merging are excluded for the next round of sampling (see the use of visit[] in the HillClimbingConfigurationSearch in Algorithm 1).

Once a segment *B_j _*is chosen, it is tentatively merged with segment *B_j+1 _*next to *B_j_*. Then a logistic regression model is re-calculated. The two segment merging is accepted only if the merging of two segments reduces the weighted squared error (equation 2). Otherwise, the original segment configuration is retained, rejecting the merging. A segment *B_j _*considered for merging is marked so that the segment will not be repeatedly chosen for the next step. This sampling and marking a segment is repeated until all segments in the current configuration are considered for merging.

**Input **: A set of pre-selected k-mers K = {*x_i_*}; Occurrences of K; Methylation levels at CpG sites

**Output**: A logistic regression model; A segment configuration.

**HillClimbingConfigurationSearch**(N)

begin

   (C*, E*, M*) = RandomConfigurationSearch ()

   **for ***i *← 2 **to ***N ***do**

      (*C*, *M*, *E*) = RandomConfigurationSearch ()

      **if ***E *<E* **then**

         C* = *C*; M* = *M*; E* = *E*

      **end**

      **report **(C*, M*, E*)

   **end**

end

**RandomConfigurationSearch **( )

begin

   *C *= InitialConfiguration (); *E *= 1.0         //Reset configuration; See text.

   **while ***true ***do**

      (C',M',E') = RandomBinaryMerging(*C*)

      **if **(*E *- *E*') ≤ *δ ***then break**

      *C *= *C'*; *M *= *M*'; *E *= *E'*

      **return ***(C,M,E)*

   **end**

end

**RandomBinaryMerging**(**configuration ***C*)

begin

   *M *= computeModel(*C*, *K *)         //Equation 1; Training stage only

   *E *= computeError(*C*, *M *)         //Equation 2

   **bool ***visit*[*n*] = **{false**}         //Mark that no segments are considered.

   **while **∃*i such that visit*[*i*] = = **false do**

      *j *= selectAtRandom(*visit*)         //See text.

      *visit*[*j*] = **true **         //*s_j _*is merge candidate.

      *C*' = *C*

      $B_{i}^{C^{\prime }}$ = **false **         //Merge*s_j _*and *s_j+1_*.

      *M*' = computeModel(*C*', *K *)         //Equation 1; Training stage only

      *E*' = computeError(*C*', *M*')         //Equation 2

      **if ***E *≤ *E*' **then**

         *C *= *C*'; *visit*[*j *+ 1] = **true **         //Accept *C*'.

      **else**

         $B_{i}^{C^{\prime }}$ = **true **         //Reject*C*'.

      **end**

   **end**

   **return ***(C,M,E)*

end

**Algorithm 1: **Hill climbing configuration search algorithm. An algorithm tries to merge two segments at random until all segments are considered for merging. A new configuration is accepted only when the error is reduced with a new logistic regression model, thus it is a hill climbing algorithm.

## Results

### Data set

We used data from Zhang et al [16] for DNA methylation patterns in chromosome 21 (297 amplicons from 190 gene promoters using bisulfite conversion, subcloning and sequencing DNA as the major experimental methods). The bisulfite sequencing data were collected in five cell types: viz. human peripheral blood (primarily leukocytes), fibroblast, the human embryonic kidney cell line HEK293, the human hepatocellular liver carcinoma cell line HepG2 and fibroblast cells derived from a patient with Down syndrome (trisom 21). Methylation patterns differed widely and specific to each cell types.

### Experimental setup

The 10-fold cross validation (described above) was used to compare the performances of three modeling approaches. For each round of 10-fold validation, one of the 10 subsets was set aside for testing, and the k-mer features were selected only from the training set, ensuring that the test data would have no influence on the k-mer feature selection. Also, regression coefficients were computed in only training stage. We measured the area under the ROC curve (AUC) score for performance comparison.

### Effectiveness of the segment modeling approach

We extensively tested the effectiveness of the segment modeling algorithm using 4-mer, 5-mer, and 6-mer patterns. For each of the experiments, the AUC score was measured from 10-fold cross validation for the initial segment definition vs. the final segment definition. The RF-based algorithm with 100 trees was used for k-mer feature selection. For each k-mer selection procedure, 30 random experiments were performed, and k-mers with z-score > 0 that appeared in at least 90% of experiments were selected as k-mer features. Using the set of k-mers, the optimal logistic regression model was computed.

### 10-fold cross validation experiments

**Figure 2:**
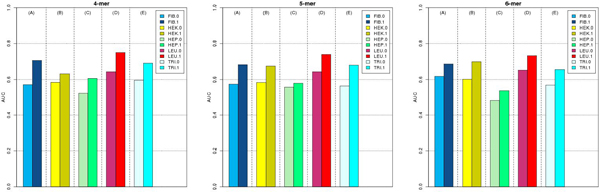
**Effectiveness of segment modeling in 10-fold cross-validation experiments.** Bars between adjacent dotted lines show improvement in the between prediction results of two models with the initial segment setting and the final segment setting in terms of AUC scores. We measured the performance improvement using 4-mer, 5-mer, and 6-mer features. For each cell type, the segment modeling algorithm identified significantly improved segment definitions. Five panels in each plot corresponds to tissue types: (A) Fibroblast, (B) HEK293, (C) HepG2, (D) Leukocytes, and (E) Trisom 21.

The performance comparison between the initial segments and the final segments in the test set is shown in Figure 2. Bars between adjacent dotted lines show the improvement in the between prediction results of two models with the initial segment setting and the final segment setting in terms of the AUC scores. We measured the performance improvement using 4-mer, 5-mer, and 6-mer features. For each cell type, the segment modeling algorithm identified significantly improved segment definitions. Five panels in each plot correspond to tissue types: (A) Fibroblast, (B) HEK293, (C) HepG2, (D) Leukocytes, and (E) Trisom 21. Our algorithm achieved approximately 10% improvement in most cell types, illustrating the effectiveness of the segment modeling algorithm.

### Search behavior

**Figure 3:**
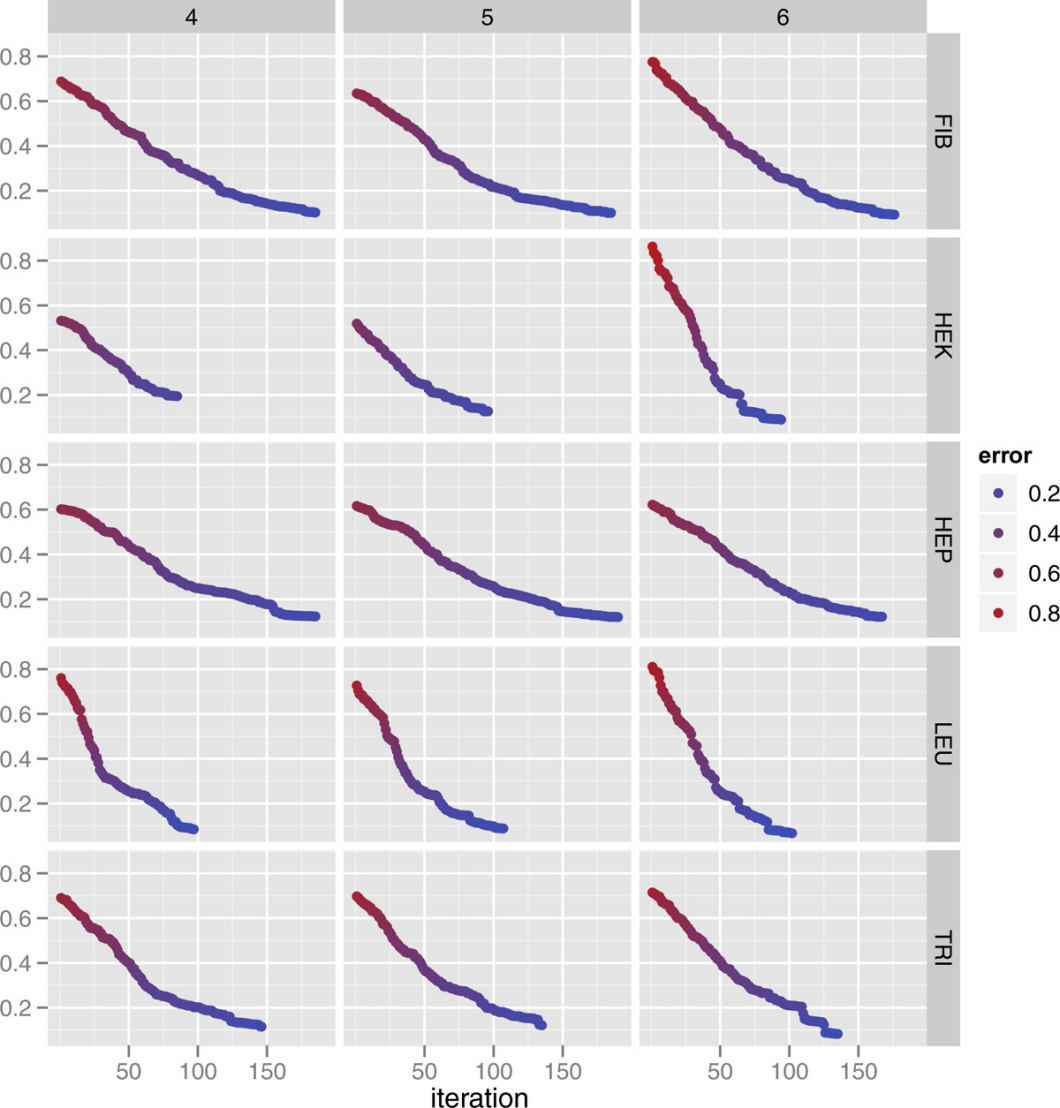
**The search behavior of the segment modeling algorithm using the whole data set.** Pairwise plots showing reduced learning error (2) at each iteration of segment merging and model recalculation. The columns for the pairwise plots are k-mers; rows are cell lines. In each plot, the X-axis denotes the number of iterations and the weighted squared prediction error is denoted on the Y-axis. The HillClimbing search algorithm effectively reduced the error between prediction and observation. In fitting the whole data set, as opposed to 10 fold cross validation, the final model predicted methylation susceptibility in the different cell types.

The search behavior of the segment modeling algorithm is shown in Figure 3. In this experiment, we used the whole data set to show the algorithmic convergence of our approach. The learning error (Equation 2) was reduced at each iteration of segment merging and model re-calculation. Our random segment sampling algorithm converged for all 15 cases of 5 different cell lines with 4-, 5-, and 6-mers.

### Discussion on the predictive power of the model

The predictive power of the model measured by 10-fold cross validation is encouraging. For 6-mers, the predictive accuracy was 0.69 for Fibroblast, 0.70 for HEK293, 0.54 for HepG2, 0.73 for Leukocytes, and 0.65 for Trisom 21. These prediction accuracies using 6-mer cannot be achieved in random data sets where the expected prediction accuracy is 0.5. Variations in the prediction accuracy for the five cell types, especially for HepG2, may be due to the cell type specific characteristics. On the other hand, the data obtained from [16] was of a low coverage. Amplicons covered less than 0.2% of entire Chromosome 21. Thus variations in the prediction accuracy may due to the low coverage of the data used. We were not able to further verify why the prediction accuracy varied. In fitting the whole data set, as opposed to 10 fold cross validation, the final model was able to accurately predict methylation susceptibility.

### Effect of the number of k-mers used for prediction

**Figure 4:**
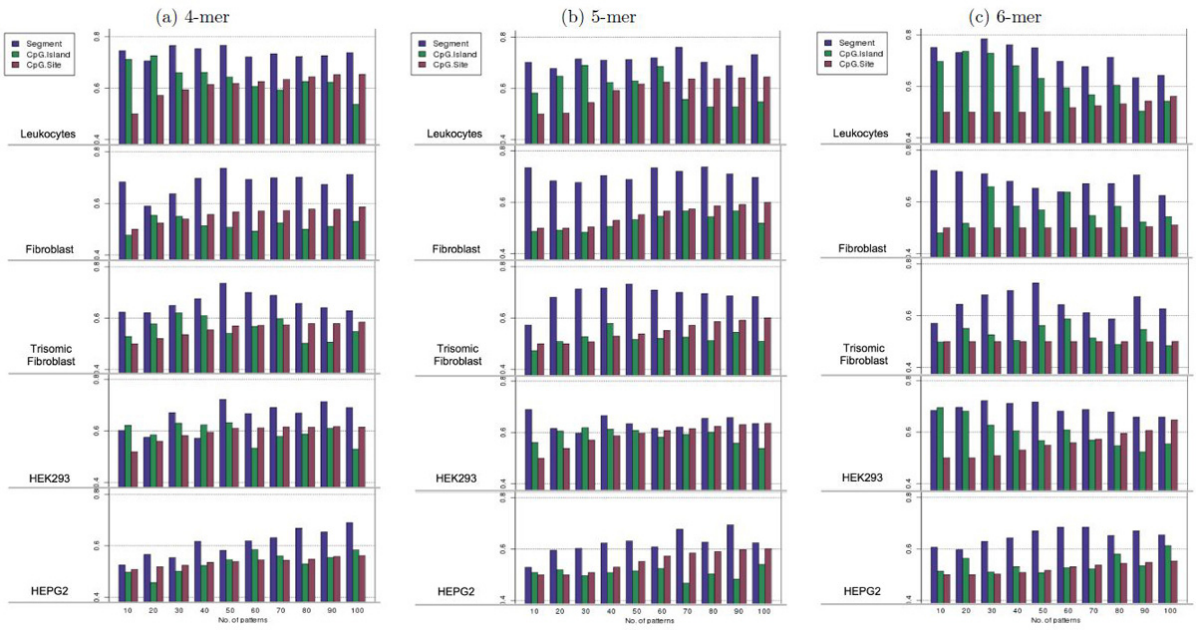
**Effect of the number of k-mers used for three modeling approaches.** The performance of three modeling approaches was measured from 10-fold cross-validation. Each bar is the AUC value of the experiment. X-axis is the number of most significant variables (p-value in t-test) used in each experiment. Consistently in 4-mer to 6-mer and regardless of number of patterns, segment modeling outperformed other modeling approaches. More importantly, from the experiments using variable numbers of k-mers from 10 to 100, we have shown that the selection of k-mers does not have a big impact on the model performances and the higher accuracies of the segment modeling approach, compared to the promoter and site-specific modeling approaches, is likely due to the effectiveness of the segment model.

The three modeling approaches were compared in terms of AUC obtained by 10-fold cross-validation technique. We conducted comprehensive modeling of cell-type specific DNA methylation susceptibility at three different resolutions: individual CpG sites, CpG segments, and promoter regions in terms of AUC obtained by the 10-fold cross validation technique. The methods for modeling at individual CpG sites and at promoter regions are described in Additional file 2. To measure the effect of the number of k-mer patterns used for modeling, 10-fold cross-validations were performed with a varying number of k-mer patterns from 10 to 100 (with an increase of 10 k-mers). P-values from t-tests were used to select the k-mers. The experimental results are illustrated in Figure 4. Only the segment modeling approach was effective for all 4-, 5-, and 6-mer experiments. Interestingly, the number of k-mers used for modeling had little impact on the prediction result, demonstrating that the prediction accuracy did not derive from the over-fitting the data and indicating that the selection of a small number of k-mers can effectively model methylation susceptibility without a loss of prediction power. Moreover, when a longer k-mer was used (up to 6-mer), the prediction accuracy did not decrease. This finding is highly encouraging because on average, a 6-mer is unlikely to occur by chance in a short (274 bp) DNA segment. Thus, a set of 6-mers can be used to model DNA methylation susceptibility.

## Conclusion

We conducted a comprehensive modeling study for cell-type specific DNA methylation susceptibility. By performing extensive computational experiments of data from five distinct cell types, we show that DNA methylation susceptibility can be accurately modeled at the segment level, achieving up to 0.75 in AUC prediction accuracy in a 10-fold cross validation study. The two-step iterative segment modeling algorithm successfully identified optimal segments that can be modeled as a logistic regression model using a set of k-mers. Our model further shows the significance of certain k-mers for the mixture model, which can potentially highlight DNA sequence features (k-mers) of differentially methylated promoter CpG island sequences in different cells and tissues, including malignancies. As only used 4 bp patterns were used in previous modeling studies of DNA methylation susceptibility, this is the first report to show that k-mer modeling can be performed using up to 6-mer without the loss of modeling accuracy.

## List of abbreviations used

• AUC: area under the ROC curve; • DNA: deoxyribonucleic acid; • MP: methylation-prone; • MR: methylation-resistant; • RF: random forest; • YY: Youngik Yang; • SK: Sun Kim; • KN: Ken Nephew.

## Competing interests

The authors declare that they have no competing interests.

## Authors' contributions

YY designed the computational framework, conducted simulation, and wrote the manuscript. KN gave critical input on biological discussion of this work, and drafted the manuscript. SK led the project, designed the algorithm and tests, and drafted the manuscript.

## Supplementary Material

Additional file 1 **DNA methylation level variation.** A figure in the file shows DNA methylation level variation in an amplicon from 5 cell types. Click here for file

Additional file 2 **Competing modeling approaches.** Compared to segment modeling, two competing modelings, CpG site-specfic modeling and promoter region modeling were described. Click here for file
