# Supplementary material for: A novel k-mer mixture logistic regression for methylation susceptibility modeling of CpG dinucleotides in human gene promoters
Source: BMC Bioinformatics. 2012 Mar 21;13(Suppl 3):S15. doi: 10.1186/1471-2105-13-S3-S15 (PMC3311103; doi:10.1186/1471-2105-13-S3-S15)
Supplement: Additional file 1 — DNA methylation level variation. A figure in the file shows DNA methylation level variation in an amplicon from 5 cell types. [file 1471-2105-13-S3-S15-S1.pdf]

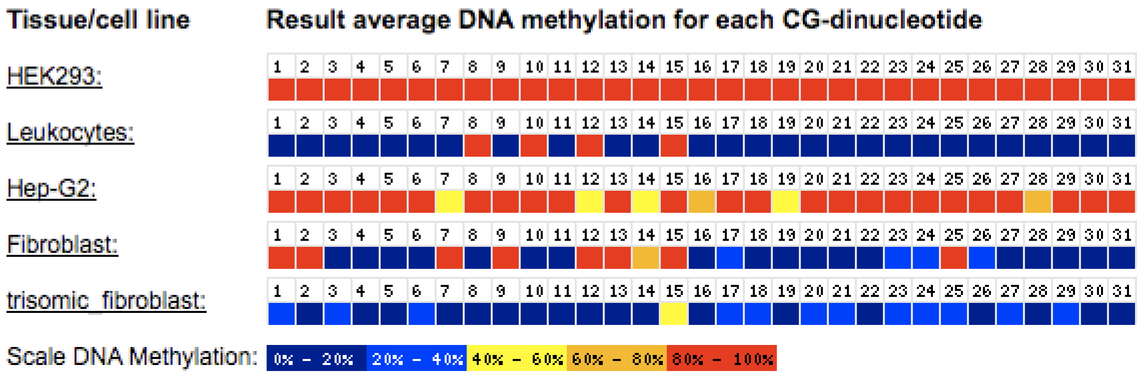

DNA methylation level variation.

The figure shows DNA methylation level variation in amplicon 193 from 5 cell types.

The region has 31 CpG sites and methylation levels are widely different among tissues regardless of the same primer region.

The figure was captured from *http://biochem.jacobs-university.de/name21/presentation/amplicon_summaries/193_amplicon_summary.html*.
