# Supplementary material for: A novel k-mer mixture logistic regression for methylation susceptibility modeling of CpG dinucleotides in human gene promoters
Source: BMC Bioinformatics. 2012 Mar 21;13(Suppl 3):S15. doi: 10.1186/1471-2105-13-S3-S15 (PMC3311103; doi:10.1186/1471-2105-13-S3-S15)
Supplement: Additional file 2 — Competing modeling approaches. Compared to segment modeling, two competing modelings, CpG site-specfic modeling and promoter region modeling were described. [file 1471-2105-13-S3-S15-S2.pdf]

1 CpG site-specific modeling

In this setting, the DNA methylation susceptibility modeling is performed at the CpG site level. The method used to calculate the methylation level at each CpG site is illustrated in Figure 1.

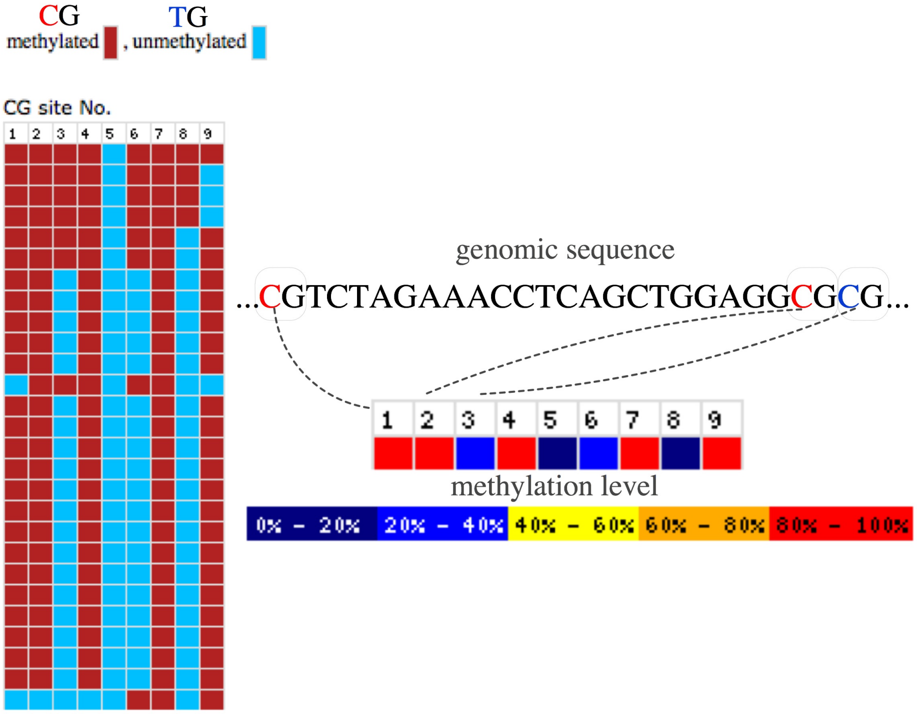

Figure 1: Illustration of CpG site specific methylation level. The 5-level methylation status scheme is categorized by [1]. CpG sites with DNA methylation status in the gray area (between 40% and 60%) were not considered, as suggested by [1]. The methylation status was then labeled as one of two categories, a methylation susceptible class (>60%) and a methylation resistant class (<40%).

Labeling data: The methylation level **p** was measured as a probability where $p_j = \#CGs/(\#CGs + \#TGs)$ at each CpG site s_j. Then, a label t_j was assigned for s_j as either $+$ or $-$ depending on its methylation levfel. A $+$ label was given to t_j where $p_j > 0.6$ and a $-$ label was assigned to t_j where $p_j < 0.4$. Any CpG site whose DNA methylation level is in gray area (between 0.4 and 0.6) was not used, as suggested by [1].

Attributes for modeling: K-mers within length w (50 by default) base pairs centered at s_j were extracted from the reference human genome sequence (*hg18*).

Modeling: Modeling DNA methylation susceptible CpG sites $\{s_j\}$ was carried out by logistic regression using attributes $\mathbf{x}$ and target $\mathbf{t}$. We built a model per each tissue type and computed a logistic model by minimizing the error between predicted and observed methylation status.

2 Promoter region modeling

Labeling data: The methylation level of a promoter region PR_k was defined as an aggregation of all CpG sites $\{s_j\}$ within PR_k. The methylation status of each site was labeled as either + or - as we did for the site-specific modeling. Then, the methylation probability p_k of PR_k was defined as a ratio of the number of CpG sites in PR_k to the number of CpG sites with the + label. The label t_k of PR_k was then assigned + if p_k is greater than 0.5. Otherwise, a label $-$ is assigned to t_k.

Attributes for modeling: K-mer occurrences in promoter regions were used as attributes. For each PR_k, we extracted a region of sequence from reference sequence (*hg18*). K-mer pattern occurrences in the methylation susceptible and not susceptible promoters were used to select a small subset of k-mers features $\mathbf{x}$.

Modeling: Given a set of k-mer patterns, a single logistic model was used to model the methylation status of all promoters, using attributes $\mathbf{x}$ and labels $\mathbf{t}$.

References

[1] Y. Zhang, et. al. "Dna methylation analysis of chromosome 21 gene promoters at single base pair and single allele resolution," *PLoS Genet*, vol. 5, no. 3, pp. e1 000 438+, 2009.
